# Duchenne muscular dystrophy newborn screening: the first 50,000 newborns screened in Taiwan

**DOI:** 10.1007/s10072-022-06128-2

**Published:** 2022-05-13

**Authors:** Yin-Hsiu Chien, Ni-Chung Lee, Wen-Chin Weng, Li-Chu Chen, Yu-Hsuan Huang, Chao-Szu Wu, Wuh-Liang Hwu

**Affiliations:** 1 Department of Medical Genetics, National Taiwan University Hospital, Taipei, Taiwan; 2 Department of Pediatrics, National Taiwan University Hospital, Taipei, Taiwan; 3 Department of Pediatrics, National Taiwan University College of Medicine, Taipei, Taiwan

**Keywords:** Duchenne muscular dystrophy (DMD), Creatine kinase (CK), Whole-exome sequencing (WES), Newborn screening (NBS)

## Abstract

**Background:**

Duchenne muscular dystrophy (DMD/Duchenne) is a progressive X-linked muscular disease with an overall incidence of 1:5,000 live male births. Recent availability in treatment for DMD raised the need of early diagnosis, and DMD became as a selective item of newborn screening (NBS) since Feb. 2021 in our center.

**Materials and methods:**

Dried blood spots (DBS) muscle-type creatine kinase (CK) isoform was measured with a commercialized kit with age-adjusted cutoffs. Subjects with an elevation of CK in the first screen were requested for a re-screen 2 weeks later. A DBS whole-exome sequencing (WES) panel for dystrophin and other neuromuscular-related genes was applied to confirm the diagnosis for subjects with persistent hyperCKemia.

**Results:**

During a 1-year period, 50,572 newborns (male 26,130) received DMD screening at a mean age of 2 days (SD 1 day). Among them, 632 (1.2%) had an elevated CK value. A re-screen at a mean age of 14 days (SD 8 days) revealed 14 subjects with persistent hyperCKemia, and DMD was confirmed in 3 of them. The incidence of DMD in Taiwan was 1:8,710 (95% CI 1 in 2,963 to 1 in 25,610) live birth males. Results of DMD DBS also assisted in Pompe newborn screening.

**Conclusions:**

NBS for DMD enables earlier management of the disease. The high re-screening rate could potentially be waived by moving the DBS WES assay to a second-tier test. The long-term benefit and the impact of newborn screening on the prognosis of DMD, however, remain further elucidated.

## Introduction

Duchenne muscular dystrophy (DMD) is an X-linked muscular degenerative disorder, leading to wheelchair confinement at 8–12 years of age and the associated cardiomyopathy, with an incidence of 1:3,600–9,300 live male births [1]. Despite advances and increased availability of genetic testing for DMD, the mean age at diagnosis is 3.5–5 years, usually up to 2 years later than the appearance of first symptoms. New treatments for DMD are also developing, including reading-through [2], exon-skipping, and gene therapy [3].

Newborn screening (NBS) for DMD by measuring creatine kinase (CK) activity has been attempted for a few years; however, a high false-positive rate was reported in all programs; therefore, variable strategies including re-screen and molecular testing have been employed [4–6]. We have added DMD NBS into our routine NBS program since 2021. Here, we report our screening strategy and results of the first year of universal, population-based screening in 50,000 newborns.

## Materials and methods

The NBS Center at the National Taiwan University Hospital (NTUH) conducts routine newborn screening for approximately 35% born babies around Taiwan. DBS samples were obtained at 48–72 h after birth. DBS CK-MM was measured using a GSP^®^ Neonatal Creatine Kinase-MM kit with a fully automated GSP instrument (both manufactured by PerkinElmer, Turku, Finland) according to the manufacturer’s instruction. Newborns who had a first-screen CK-MM level higher than cutoffs (the 99^th^ percentile of normal newborns, 750 ng/mL whole blood (WB) in full term babies and 650 ng/mL in premature babies) were requested to have a re-screen 2 weeks later. The cutoff for re-screen was 300 ng/mL. For those with an elevated CK-MM level at re-screen, defined as persistent hyperCKemia, a 3^rd^ CK-MM measurement and molecular analysis were requested. The molecular analysis was performed on DNA extracted from DBS using a whole-exome sequencing (WES) muscle panel for the targeted DMD gene and other neuromuscular related genes.

## Results

**Fig. 1:**
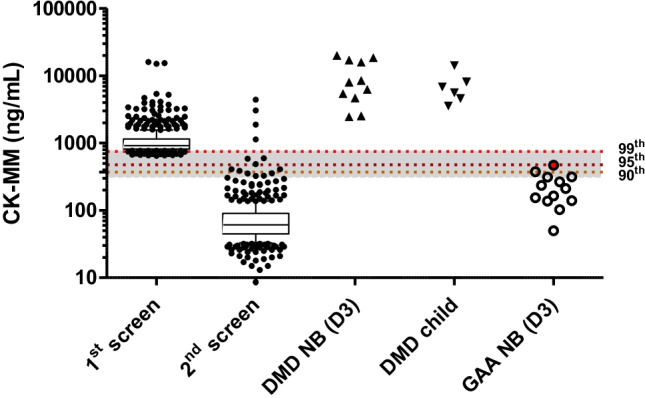
Dried blood sample (DBS) CK-MM levels in different groups of subjects. Newborns who had a CK-MM level higher than the cutoffs at first screen (1^st^ screen, *n*=632) were recalled 2 weeks later (2^nd^ screen, *n*=510). CK-MM levels at the second screen were normal in all cases except in 14 subjects. Archived NBS samples from 11 male patients with DMD (DMD NB) and DBS samples from 6 patients at the age of 2–13 years (DMD child) were used as comparisons. First screened DBS CK-MM levels from newborns with abnormal Pompe NBS results (GAA NB) were plotted, and the one with infantile-onset Pompe disease was shown in circle filled with red. The boxes indicated the 1–99^th^ percentile values in each group, and the bar inside the box was the median of each group. The dash lines indicate the 99^th^, 95^th^, and 90^th^ percentiles of the normal controls of the first screen, respectively

Between Feb. 2021 and Dec. 2021, 50,572 newborns were screened with the CK-MM assay at a mean age of 3 days (SD 3 days). In total, 1.2% (632) of the newborns displayed an elevation in CK-MM in their first samples collected at a mean age of 2 days (SD 1 day), slightly earlier than the population’s practice. Among them, 510 (81%) re-call samples were tested at the mean age of 12 days (SD 8 days) and were returned to normal CK-MM levels. Five subjects (1%) had died of unrelated reasons and were not be able to have another check. Four subjects (one female and three males) were unable to do the re-screen, and WES using the first-screen DBS revealed a variant on *COL12A1* gene in a male subject. Fourteen newborns (2%) met the criteria of persistent hyperCKemia, but eleven (7 males and 4 females) of the 14 newborns had normal CK-MM levels in their 3^rd^ samples. Molecular tests in the remaining 3 subjects with persistent hyperCKemia confirmed as having variants on *DMD* gene (Fig. 1) in all of them. The first CK-MM values for cases 1, 2, and 3 were 5247, 15983, and 15104 ng/mL, respectively. The 2^nd^ CK-MM values were 1885, 3058, and 4403 ng/mL, respectively. Finally, the serum CK levels as the confirmatory tests were 4070, 5337, and 3457 U/L, respectively.

Two subjects (cases 1 and 2), one with an approximately 600-bp deletion involving exon 20 of the *DMD* gene and the other involving 8 exons deletion from exon 44 to exon 51, inherited the deletion from the mothers, but no other affected individuals known in the families. The third subject had a single nucleotide variation c.9337C>T (p.R3113*), which should cause a premature termination of the transcript. Overall, the incidence of DMD derived from the current study was 1:8,710 (95% CI 1 in 2,963 to 1 in 25,610) live birth males. Case 1 showed a borderline below gross motor performance quotient at age 6 months, and the use of Deflazacort was under discussion.

**Fig. 2:**
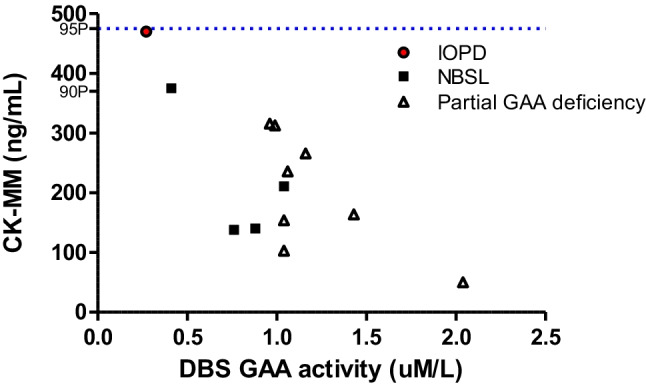
DBS CK-MM levels in subjects with positive Pompe disease screening. In total, 13 newborns showed low in acid alpha glucosidase (GAA) in the dried blood spots (DBS), defined as positive in Pompe newborn screening, were plotted. The one with infantile-onset Pompe disease (IOPD) had the lowest DBS GAA activity, and a high CK-MM level at the 95^th^ percentile (blue dash line). Only one of the four newborns (NBSL) with GAA deficiency but no cardiomyopathy, defining as later-onset Pompe disease, had a CK-MM levels at the 90^th^ percentile. All the others (partial GAA deficiency), although low in DBS GAA activity, were diagnosed as partial GAA deficiency by their genotypes and lymphocytes GAA activities and they all have quite normal DBS CK-MM levels.

We hypothesized that first-screen CK-MM levels could help make the clinical judgement of babies with acid alpha glucosidase (GAA) deficiency or Pompe disease. GAA deficiency was defined as lymphocyte GAA activity <3% of the normal mean and GAA variants on both the maternally and paternally inherited chromosomes; infantile-onset Pompe disease (IOPD) refers to patients with cardiac involvement at the time of diagnosis, and all other cases are classified as later-onset Pompe disease and were followed for symptoms as previously reported [7]. In this period, we encountered 13 subjects with positive Pompe NBS, with 5 GAA deficiency and 8 partial GAA deficiency. Among them, only 1 newborn with CK-MM at 470 ng/mL (Fig. 1 and 2), approximately at the 95^th^ percentile of the newborn range, had the lowest GAA activity in DBS, and was confirmed as a patient with classical IOPD due to the presence of hypertrophic cardiomyopathy. We have initiated the disease-specific treatment at the age of 9 days. The 2^nd^ highest CK-MM, 375 ng/mL, was approximately at the 90^th^ percentile and was found in a newborn diagnosed with later-onset Pompe disease. The other 3 newborns with later-onset Pompe disease and the other 8 newborns with partial GAA deficiency had normal CK-MM levels. None of the 4 subjects with later-onset Pompe disease has been on treatment yet. During this period, we did not encounter newborns who were positive for spinal muscular atrophy (SMA) screening; therefore, there was no information about the utility of CK-MM assay in SMA NBS.

## Discussion

Here we reported a universal NBS for DMD using a FDA-approved immunoassay. In contrast to a re-screen rate of 0.9% (237 out of the 26,135 newborns) in the New York State (NYS) Newborn Screening Program [8], we experienced a high intended re-screen rate (1.2%), in order not to have false negatives as in the Wales program [9] or miss boys with a Becker muscular dystrophy phenotype [4, 5]. Most subjects have normal CK-MM levels at the second screen at 2 weeks of age, because the CK-MM levels indeed stabilized at 1 weeks of life [8]. We could improve the specificity of DMD NBS by adding a 2^nd^-tier WES test when the original samples revealed high CK-MM [5, 10]; this will be especially benefit to those who were not able to have a 2^nd^ sample, say, in the period of COVID-19 pandemic as in our current report, and to whom had excessive stress facing the potential false-positive results. However, the add-on cost was not acceptable in our current program and was not executed yet.

In the current report, we identified newborns with DMD in one out of 8,710 live male newborns. Recent advances such as the increased disease awareness, uptake of expanded carrier screening, and the usage of advanced prenatal testing all should have an impact to the nature occur incidence of DMD. Nevertheless, one recent genotype study in Taiwan showed that only 50% of DMD cases could be detected by multiplex ligation-dependent probe amplification (MLPA) [11], implying the limitation of current carrier or prenatal DMD screening. NBS using a biochemical marker will be necessary for early diagnosis of DMD.

DMD NBS process additional utilities as demonstrated in our report. Since other diseases with the associated CK-MM elevations may be revealed by this, it is useful to conjunction DBS CK-MM to other screening such as Pompe NBS to help the identification of IOPD so that treatment can be start immediately. Only patients with IOPD, but not LOPD, will have the elevation in CK-MM at the first screening sample.

A clear guideline for prospectively followed asymptomatic DMD patients on when and how to initiate treatment is not available yet. Most of the clinical trials for new treatment were conducted in patients aged 4 years or older, even though patients may present with gross motor delay from 4 to 4 months of age [12]. For the use of glucocorticosteroids, only Deflazacort has been approved for patients aged 2 years and older. Long-term outcome data generated from newborn screening will certainly be critical to make a guideline for pre-symptomatic follow-up of DMD.
